# Left ventricular motion quantification parameters from tissue phase mapped MRI: influence of gender

**DOI:** 10.1186/1532-429X-17-S1-Q20

**Published:** 2015-02-03

**Authors:** Jan Paul, Raphael Beck, Dominik Buckert, Peter Bernhardt, Wolfgang Rottbauer, Heiko Neumann, Volker Rasche

**Affiliations:** 1 Internal Medicine II, University Hospital of Ulm, Ulm, Germany; 2 Institute of Neural Information Processing, University of Ulm, Ulm, Germany

## Background

Several motion parameters for quantification of LV motion abnormalities and asynchrony have been described in literature, but were investigated with different acquisition protocols and for different cohorts, thus impeding comparability. For derivation of normal values in healthy volunteers, calculation of all parameters from a single acquisition protocol is desirable. In this study, influence of gender on the motion parameters is investigated in order to determine the necessity of separate female/male normal values.

## Methods

### Cohorts and acquisition

20 females (27.3±6.6 y) and 21 males (23.6±2.1 y) without known cardiovascular diseases were investigated. Acquisition parameters were: Philips Achieva 3 T, 32 channel cardiac coil, velocity encoded (Tissue Phase Mapped, TPM) segmented black-blood gradient echo with VENC=30 cm/s, TR/TE=6.1/4.6 ms, FOV adapted to patient size, resolution = 2x2x8 mm^3^, 3 k-lines/segment, SENSE=2, phase interval=30 ms, and nominal scan time=5:51 min:sec for 3 short axis slices.

### Motion-Quantification parameters (see [1])

The following parameters were calculated:

a) velocity-based: Standard Deviation of Times to Peak [σ(TTP)], Asynchrony Correlation Coefficient [ACC], Temporal Uniformity of Velocity [TUV], and difference of peak velocities [Δv].

b) rotation-based: Base Apex Rotation Correlation [BARC].

c) strain-based: Temporal Uniformity of Strain [TUS], Standard Deviation of Onset/Peak Time [σ(T_onset/peak_)], Coefficient of Variation [CV], Difference between Septal and Lateral Peak Circumferential Strain [DiffSLpeakCS], Onset/Peak Of Shortening Delay [OS/PS Delay], Regional Variance of Strain [RVS], and Regional Variance Vector of Strain [RVVPS].

### Analysis

Difference of these parameters between the female and male group was assessed by a non-parametric Wilcoxon rank-sum test and p-values below 5% were considered significant.

## Results

**Figure 1:**
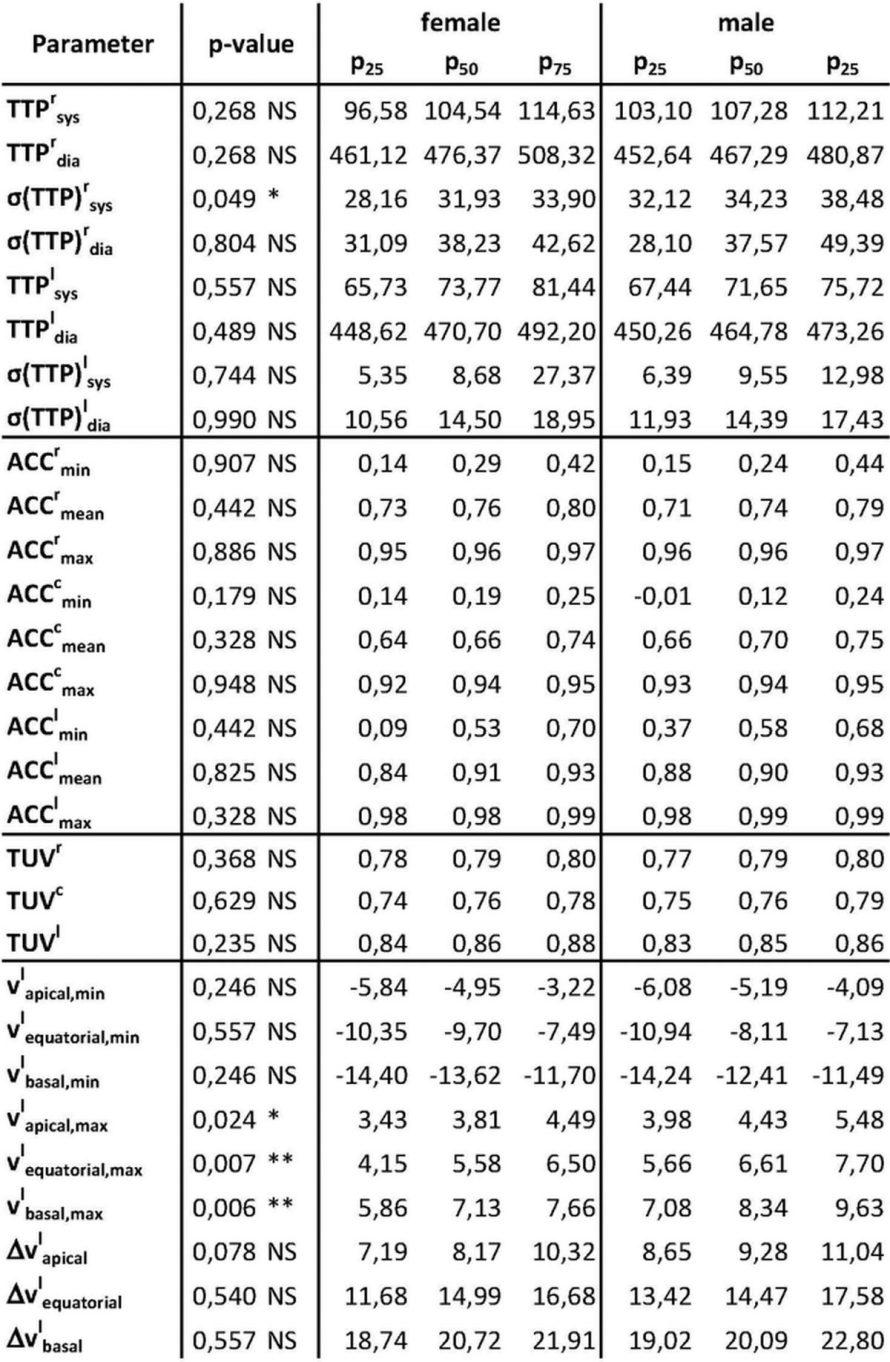
P-values and significances for comparison of the female vs. male group for the different motion quantification parameters. Also shown are lower (p_25_) and upper quartile (p_75_) and the median (p_50_). Abbreviations: r = radial, c = circumferential, l = longitudinal; ***: p < 0.001, **: p < 0.01, *: p < 0.05, NS: p >= 0.05.

**Figure 2:**
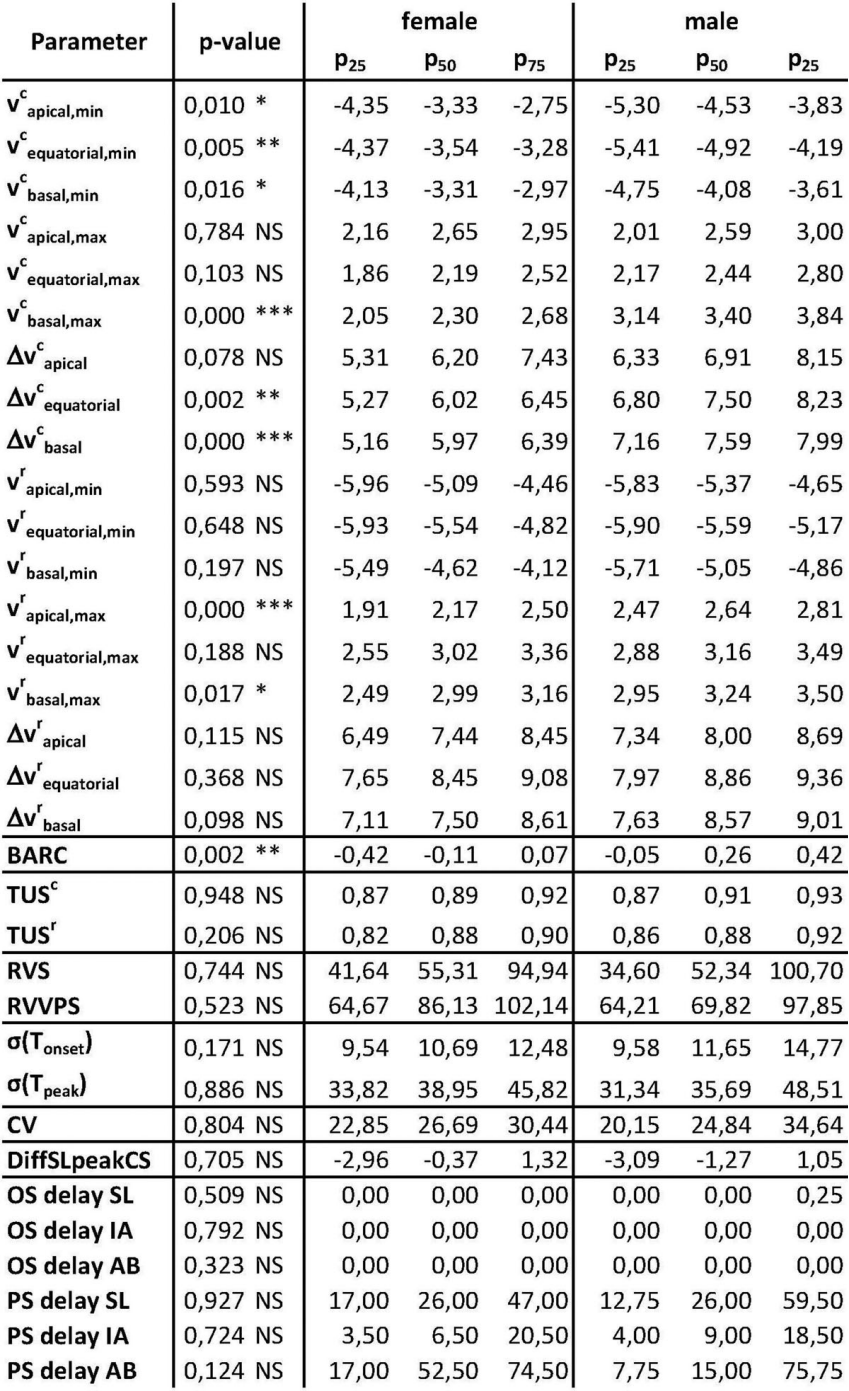
(continued from Figure 1). Abbreviations: SL = septal-lateral, IA = inferior-anterior, AB = apical-basal.

Significant differences were found for peak velocities v^l^_max_ and v^c^_min_ in all slices and v^c^_basal,max_, v^r^_apical,max_, v^r^_basal,max_ in individual slices, as well as the peak velocity differences Δv^c^_equatorial_ and Δv^c^_basal_, and σ(TTP)^r^_sys_. The rotation-based parameter BARC showed also significant differences. All other differences were not statistically significant (see Figures 1 and 2).

## Conclusions

While peak velocities and their difference Δv as well as BARC require separate consideration for males and females, all velocity and strain based asynchrony parameters except σ(TTP)^r^_sys_ show no dependence of gender and thus appear suitable to calculate normal values from a mixed female/male healthy volunteer cohort.

## Funding

This work is partly funded by a research grant from Philips Healthcare.
